# Protective Effect of *Cicer arietinum* L. (Chickpea) Ethanol Extract in the Dextran Sulfate Sodium-Induced Mouse Model of Ulcerative Colitis

**DOI:** 10.3390/nu12020456

**Published:** 2020-02-12

**Authors:** Mia Kim, Kyung-Sook Chung, Se-Jung Hwang, Ye Seul Yoon, Young Pyo Jang, Jong Kil Lee, Kyung-Tae Lee

**Affiliations:** 1 Department of Cardiovascular and Neurologic Disease (Stroke Center), College of Korean Medicine, Kyung Hee University, Seoul 02447, Korea; hyuntemia@hanmail.net; 2 Department of Pharmaceutical Biochemistry, College of Pharmacy, Kyung Hee University, Seoul 02447, Korea; adella76@hanmail.net (K.-S.C.); teuerjung@naver.com (S.-J.H.); 3 Department of Basic Pharmaceutical Science, College of Pharmacy, Kyung Hee University, Seoul 02447, Korea; sri88@khu.ac.kr; 4 Department of Life and Nanopharmaceutical Sciences, Graduate School, Kyung Hee University, Seoul 02447, Korea; ypjang@khu.ac.kr; 5 Department of Oriental Pharmaceutical Science, College of Pharmacy, Kyung Hee University, Seoul 02447, Korea; 6 Department of Pharmacy, College of Pharmacy, Kyung Hee University, Seoul 02447, Korea; jklee3984@khu.ac.kr

**Keywords:** *Cicer arietinum*, colitis, dextran sodium sulfate, NF-κB, STAT3

## Abstract

Inflammatory bowel disease (IBD) is a major risk factor of colorectal cancer. Drugs currently used for IBD exhibit adverse effects including vomiting, nausea, and diarrhea. Naturally derived novel alternative therapies are required to overcome these limitations. In this study, we investigated the protective effects of ethanol extract of *Cicer arietinum* (CEE) in a dextran sodium sulfate (DSS)-induced mouse model of colitis. CEE markedly improved DSS-induced clinical symptoms and histological status, such as the disease activity index, spleen weight, and colon length. Moreover, CEE-treated mice showed significant recovery of DSS-induced crypt damage and cell death. CEE suppressed myeloperoxidase (MPO) activity and macrophage marker F4/80 mRNA expression in colonic tissue of mice with DSS-induced colitis, indicating neutrophil infiltration and macrophage accumulation, respectively. Although DSS upregulated pro-inflammatory mediators and activated transcription factors, CEE downregulated the mRNA expression of cytokines including interleukin-6, interleukin-1β, and tumor necrosis factor-α, protein expression of cyclooxygenase-2 and inducible nitric oxide synthase, as well as activation of nuclear factor-kappa B (NF-κB) and signal transducer and activator of transcription 3 (STAT3). Hence, our findings reveal that the anti-inflammatory properties of CEE, involving the downregulation of the expression of pro-inflammatory mediators by inactivating NF-κB and STAT3 in DSS-induced colitis mice.

## 1. Introduction

The integrity of the epithelial barrier is crucial for intestinal homeostasis. Barrier integrity disruption leads to an abnormal immune response and consequently results in autoimmune conditions, including inflammatory bowel disease (IBD) [1]. IBD causes instability of the human gut and an uncontrolled inflammatory response, including ulcerative colitis (UC) and Crohn’s disease [2]. UC is more common in the West, but in recent years, the global increase in IBD is believed to be associated with industrialization, and this disease is a serious health concern [3]. Active UC occurs typically in the colon and rectum, and mainly manifests as abdominal pain, bloody diarrhea with or without mucus, and weight loss. Although UC is related to genetic factors, environmental factors, infection, dysbacteriosis, and immune factors, the pathogenesis of UC is still unclear [4]. Among those factors, the immune component involving cytokine-driven mixed inflammatory infiltrate in the intestinal mucosa is the most widely accepted factor that has been investigated [5].

Dysregulation of the intestinal epithelial barrier is a consequence of inflammatory response due to the activation of transcription signaling factors, such as nuclear factor-kappa B (NF-κB) and signal transducer and activator of transcription 3 (STAT3) [6]. These transcription factors, which were initially identified as acute inflammatory phase response factors, play major roles in determining intestinal epithelial reconstitution during colitis. After inflammatory ligands bind to specific receptors, NF-κB and STAT3 are phosphorylated, resulting in translocation to the nucleus, where they bind DNA and regulate the transcription of inflammatory genes leading to pathogenic inflammatory processes [7,8]. These reports reflect the major role of transcription factors in controlling the expression of pro-inflammatory mediators, which are directly involved in the mucosal tissue damage that occurs in UC. Consequently, agents that effectively inhibit NF-κB and STAT3 activation and the subsequent expression of inflammatory mediators would be helpful for treating UC.

*Cicer arietinum* L., also called chickpea, is an annual herbaceous legume and it is one of the most widely consumed legumes in the world [9]. It is also a staple food crop that is widely grown in many tropical and subtropical countries. *C. arietinum* is known to be rich in lysine, but limited in sulfur-containing amino acids, such as methionine, tryptophan, and cysteine [10,11]. It has been reported that *C. arietinum* possesses antioxidative, estrogenic, insecticidal, piscicidal, antifungal, antimicrobial, hepatoprotective, anticancer, anti-inflammatory, and many other pharmacological effects that contribute to its health benefits [9]. However, no report has examined its protective effect against UC or the molecular mechanisms involved. Therefore, we investigated the molecular mechanisms underlying the anti-colitic properties of *C. arietinum* in a DSS-induced mouse model of colitis. 

## 2. Materials and Methods 

### 2.1. Preparation of C. arietinum Ethanol Extract (CEE)

The powder of *C. arietinum* (124.1426 g) was extracted three times with 30% ethanol (as 10 times concentration) with ultra-sonication. The extract was filtered with a filter paper (Hyundai Micro Co, Ltd., Seongnam, Korea) and freeze-dried to get 30% ethanol extract (12.4153 g; yield 10.0%).

### 2.2. Sample Preparation and HPLC-PDA-ESI-MS Analysis

The 30% ethanolic extract of Chickpea (*Cicer arietinum*, Leguminosae) was dissolved in 30% ethanol to prepare final concentration of 50 mg/mL stock solution (final concentration of ethanol is 30%). The sample was filtered through a 0.2 µm ployvinylidenefluoride (PVDF) syringe filter (Whatman International Ltd., c Kent, UK) before being injected.

A Waters Alliance 2690 HPLC system (Waters Corp., Milford, MA, USA) linked with a photodiode array detector (PDA 2996 detector, Waters Corp.) and JMS-T100TD (AccuTOF-TLC) (JEOL Ltd., Tokyo, Japan) spectrometer equipped with electrospray ionization (ESI) source were used for chromatographic and spectrometric (MS) analysis. The chromatographic separation was carried out on the Waters Atlantis^®^ T3 column (250 × 4.6 mm id, 5 μm particle size) (Waters Corp., Milford, USA). The mobile phase consisted of acetonitrile (solvent A) and acidified water with 0.1% formic acid (solvent B). The gradient condition of the mobile phase was 0–10 min, 0%; 10–22 min, 0% to 5%; 22–35 min, 5%; 35–45 min, 5% to 6%; 45–50 min, 6% to 20%; 50–60 min, 20%; 60–70 min, 20% to 22%; 70–75 min, 22% to 50%; 75–80 min, 100%; 80–105 min, 100% as percent of solvent A. The injection volume was 10.0 µL. The flow rate was 1.0 mL/min and the column oven temperature was maintained at 25 °C and the detection wavelength was 254 nm. The conditions of MS analysis in the positive ion mode were as follows: scan range, *m*/*z* 50–1000; desolvating chamber temperature, 250 °C; orifice 1 temperature, 80 °C; orifice 1 voltage, 80 V; orifice 2 voltage, 10 V; ring lens voltage, 5 V; peak voltage, 1000 V; detector voltage, 1900 V; nitrogen gas flow rate, 1.0 L/min (nebulizing gas) and 3.0 L/min (desolvating gas).

### 2.3. Animals

The male ICR mice (5-week-old, 18–22 g) were purchased from Orient Bio (Seoul, Korea). Animal welfare and experiments were carried out in accordance with the Kyung Hee University guidelines and were approved by the ethical committee for Animal Care and Use of Kyung Hee University (KHUASP(SE)-17-148, 18/01/2018).

### 2.4. Induction of Colitis

**Figure 1:**
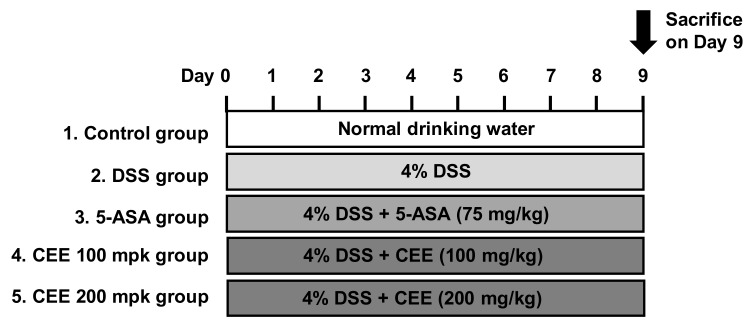
Design of animal experiments.

Colitis was induced by the administration in ICR mice with drinking water dissolving 4% (*w*/*v*) DSS for 9 days (ad libitum) (Figure 1). For each experiment, the mice were administrated orally with different material once a day for 9 days; (1) Control group: mice drank normal water and were fed vehicle (0.9% saline); (2) DSS group: mice drank DSS-containing water and were fed vehicle; (3) 5-aminosalicylic acid (5-ASA) group (positive control): mice drank DSS-containing water and were administrated 75 mg/kg/day of 5-ASA; (4) CEE 100 mpk group: mice drank DSS-containing water and were administrated 100 mg/kg/day of CEE; (5) CEE 200 mpk group: mice drank DSS-containing water and were administrated 200 mg/kg/day of CEE. All tested materials were dissolved in 0.9% saline, and the administration of each materials was started at the same time as the DSS treatment.

### 2.5. Disease Activity Index (DAI) Measurement

The body weight loss, stool consistency and the presence of gross blood in feces and at the anus were evaluated with DAI (Table 1). The body weight loss was calculated as the percent difference between the original body weight (day 0) and the body weight on the last day. In addition, the following parameters were used for calculation; stool consistency (0 points = normal, 2 points = loose stools, 4 points = watery diarrhea); hematochezia (0 points = no bleeding, 2, slight bleeding, 4 points, gross bleeding). The mice were sacrificed on the day 9 of experiments, and the distal colonic tissues and spleen were quickly collected. The colon length was measured between the proximal colon and the rectum.

### 2.6. Hematoxylin and Eosin (H&E) Staining

DSS-induced colitis appears to be more severe in the distal colon, although it causes an increase in the production of all proinflammatory cytokines in both midcolon and distal colon [12,13]. In this regard, at day 9 of DSS intake, mice were sacrificed, colons were washed and cleaned with gentle rinsing by 1× PBS to remove all the feces, distal colon fragments (~0.5 cm long) were fixed with 4% paraformaldehyde overnight. After washing with PBS, the tissue was embedded in paraffin. Tissue section and H&E staining were performed by Korea Experimental Pathology Inc. (Gyeonggi-do, Korea) [13]. For the histopathological analysis, tissue sections were made from the representative region of the distal colon using conventional tissue preparation methods, and the samples were viewed under a light microscope (400×) after H&E staining [14].

### 2.7. Western Blot Analysis

Distal colonic tissue was lysed in protein extraction buffer (PRO-PREP^TM^, Intron Biotechnology, Seoul, Korea) and the protein concentration was determined by Bio-rad protein assay reagent, following the manufacturer’s instruction. The 30 μg of protein was separated by 8–15% SDS PAGE, and transferred to a PVDF. After incubating with 5% skim milk in Tween 20/Tris-buffered saline (TTBS) at 25 °C for 1 h, the membranes were incubated with primary antibody at 4 °C for 18 h. Membranes were washed three times with TTBS and incubated with a horseradish peroxidase-conjugated secondary antibody for 2 h at 25 °C, rewashed thrice with TTBS. Blots were visualized using enhanced chemiluminescence detection agents (Amersham, Buckinghamshire, UK). 

### 2.8. Myeloperoxidase (MPO) Activity Assay

For the determination of MPO accumulation, distal colonic tissue (50 mg) was homogenized in the appropriate amount lysis buffer [50 mM potassium phosphate (pH 6.0, 0.5% hexadecyltrimethylammonium bromide)], centrifuged at 15,000 g for 10 min, and then the supernatant was evaluated by the o-dianisidine method.

### 2.9. Quantitative Real-Time Reverse-Transcriptase Polymerase Chain Reaction (qRT-PCR)

RNA was extracted from the distal colon tissue using Easy Blue^®^ kits (Intron Biotechnology, Seoul, Korea). From each sample, 500 ng of RNA was reverse-transcribed by TOPscript^™^ RT Dry MIX, and qRT-PCR amplification was carried out by the incorporation of TB GreenTM Premix Ex Taq (TaKaRa) in accordance with the manufacturer’s recommended protocols. The primer sequences are listed in Table 2. A dissociation curve analysis of genes showed a single peak for each. The mean Ct of the target gene was calculated from triplicate measurements and was normalized with the mean Ct of β-actin.

### 2.10. Statistical Analysis

Data are represented as the mean ± SDs (*n* = 10). The significance of differences was determined by the Student’s *t*-test for two-group comparisons using GraphPad Prism 5.01 software and *P*-values of less than 0.05 were reputed statistically significant. For DAI scores, statistical significance of differences was assessed with a nonparametric MannWhitney *t*-test. 

## 3. Results

### 3.1. Identification of Phytochemical Composition by HPLC-PDA-ESI-MS in CEE 

**Figure 2:**
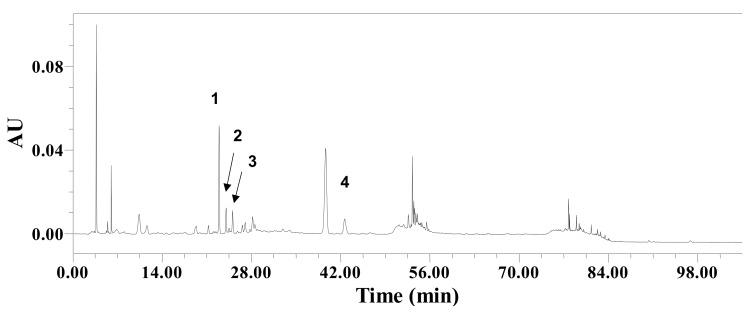
HPLC chromatogram of (*C. arietinum*) extract detected at 254 nm.

The HPLC chromatogram of CEE monitored at 254 nm is shown in Figure 2. Four compounds were identified by comparing the UV spectra and mass data from each peak of HPLC-PDA-ESI-MS with previously reported data. The retention time, precursor ion, and molecular formula of each compound are listed in Table 3. Peaks 1, 2, and 3 were confirmed as adenosine, guanine, and guanosine, respectively [15]. Peak 4 was confirmed as tryptophan [16].

### 3.2. CEE Administration Recovered the Symptoms of DSS-Induced Colitis Mice

**Figure 3:**
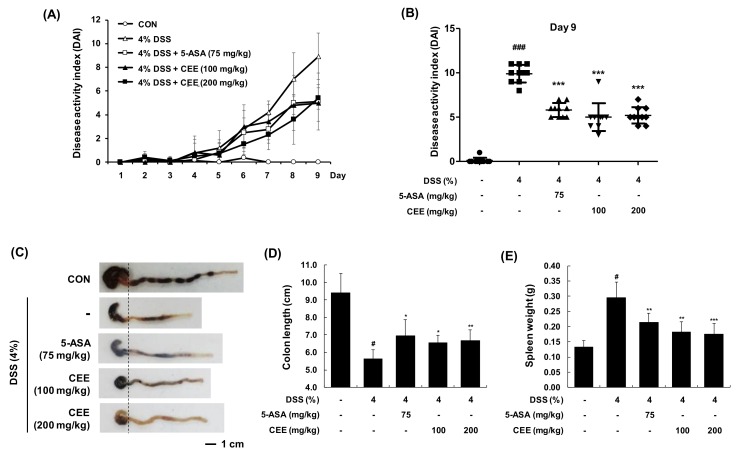
Effects of CEE on DSS-fed mice with colitis. (**A**) Disease activity index (DAI) levels during total experiments periods and (**B**) at the end of the experiment (day 9). Data are presented as mean ± SDs (*n* = 10). ^###^*p* < 0.001 vs. the vehicle-fed control group; ^***^p < 0.001 vs. the DSS-fed colitis group in a Mann-Whitney nonparametric *t*-test, 95% confidence. (**C**) Macroscopic images of the colon in each group were observed and (**D**) colon length was measured. (**E**) Experimental animals’ spleen weights. Data are presented as mean ± SDs (*n* = 10). *^#^p* < 0.001 vs. the vehicle-fed control group; ^*^p < 0.05, ^**^p < 0.01, ^***^p < 0.001 vs. the DSS-fed colitis group.

To evaluate the anti-inflammatory effect of CEE in DSS-induced colitis, we determined the changes in DAI, colon length, and spleen weight. While DAI of DSS-treated mice were significantly increased at the end of the experiment compared with those of the vehicle-treated control group, DSS + CEE (100 or 200 mg/kg, p.o.) effectively prevented the changes of DAI levels (Figure 3A,B). The total colonic length was significantly shorter in the DSS group than that in the vehicle-treated control group (9.43 ± 1.09 cm vs. 5.64 ± 0.52 cm, *p <* 0.001), whereas CEE treatment inhibited the DSS-induced colon shortening (CEE 100 mg/kg: 5.64 ± 0.52 cm vs. 6.57 ± 0.40 cm, *p* < 0.05; CEE 200 mg/kg: 5.64 ± 0.52 cm vs. 6.68 ± 0.61 cm, *p* < 0.01, Figure 3C,D). and spleen weight (Figure 3B). In addition, DSS-increased spleen weight was reduced significantly by CEE treatment.

### 3.3. CEE Ameliorated Histological Damage in DSS-Induced Colitis

**Figure 4:**
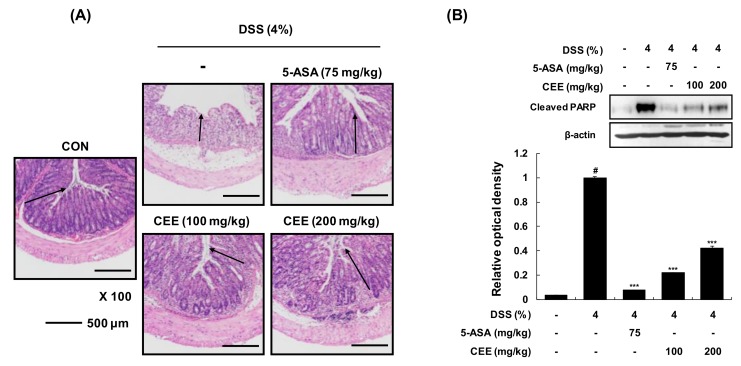
Effects of CEE on colon histology in DSS-fed colitis mice. After administration with or without CEE (100 or 200 mg/kg/day p.o.) for 9 days, (**A**) the histological changes were analyzed by H&E staining. Crypt damage was observed by Olympus cellSens Standard image software (black arrow). (**B**) Cleaved PARP levels were examined by western blot analysis using whole protein lysates from colonic tissue. *β*-Actin was used as an internal control. The ratio of relative optical density was calculated by a densitometric analysis program (Bio-Rad Quantity One^®^ Software), normalized to the internal control. Data are presented as mean ± SDs of three independent experiments. *^#^p* < 0.001 vs. the vehicle-treated control group; ^*^p < 0.05, ^**^p < 0.01, ^***^p < 0.001 vs. the DSS-fed colitis group.

The symptoms of DSS-induced colitis would be induced by direct hyperosmotic injury to epithelial cells leading to cell death [17]. In this sense, the programmed cell death machinery is key for the homeostasis reestablishment after an acute insult, limiting the propagation of the inflammatory stimuli to prevent tissue’s loss of function. After H&E staining with collected distal colonic tissue, we observed that colon tissue sections from DSS-treated mice showed irregular crypts, goblet cell loss, and moderate inflammatory cell infiltration in the submucosa. In contrast, DSS + CEE administration (100 or 200 mg/kg, p.o.) protected against damage to the colonic structure and goblet cell loss, and slightly reduced inflammatory cell infiltration (Figure 4A). To demonstrate the excessive cell death causes mucosal damage, we examined protein expression levels of the apoptotic cell death marker, cleaved poly (ADP ribose) polymerase (PARP) using western blot analysis. The protein levels of cleaved PARP were elevated in colonic tissue of DSS-treated mice, whereas administration with DSS + CEE (100 or 200 mg/kg) markedly reduced the protein level of this apoptotic cell death protein (Figure 4B). These findings suggest that CEE can alleviate DSS-induced damage to colonic tissue, including crypt changes and apoptotic cell death. 

### 3.4. CEE Attenuated mRNA Expression Levels of Cytokines in DSS-Treated Mice Colonic Tissue 

**Figure 5:**
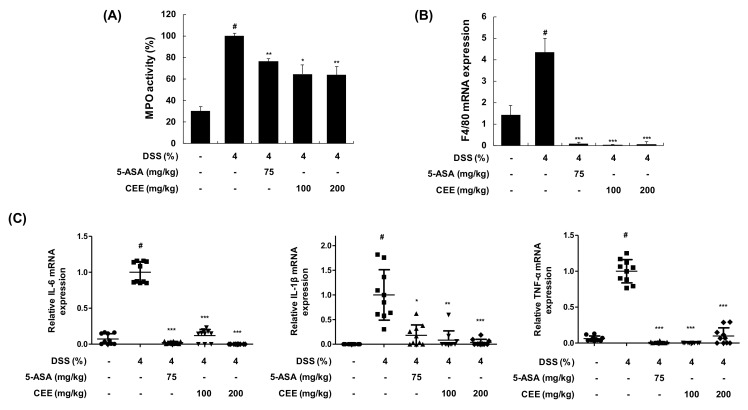
Effects of CEE on inflammatory response in mice with DSS-induced colitis. After administration with or without CEE (100 or 200 mg/kg/day p.o.) for 9 days, (**A**) colon tissues from mice were used to determine MPO activity. (**B**, **C**) The mRNA expression was determined by qRT-PCR from DSS-fed intestinal colon tissue. Using specific primers, the mRNA expression of (**B)** F4/80 and (**C**) IL-6, IL-1β, and TNF-α was determined and normalized to β-actin expression. Data are presented as mean ± SDs (*0* 10). *^#^p* < 0.001 vs. the control group; ^*^p < 0.05, ^**^p < 0.01, ^***^p < 0.001 vs. the DSS-fed colitis group.

In the DSS-inflamed colon, various immune cells are involved in secreting cytokines that regulate the inflammatory response [18]. MPO is a granule-associated enzyme present in neutrophils and widely used as a marker of intestinal inflammation [19]. To investigate the effect of CEE on the accumulation of immune cells and pro-inflammatory cytokines, we determined the MPO activity, an index used to evaluate neutrophil infiltration and mRNA expression of macrophage markers and pro-inflammatory cytokines in colon tissue from DSS-treated animals. As shown in Figure 5A, CEE treatment (100 or 200 mg/kg, p.o.) significantly suppressed DSS-induced MPO activity, indicating that CEE reduced neutrophil accumulation in colitic tissue. In addition, CEE administration reduced mRNA expression levels of F4/80 in DSS-treated colonic tissue, suggesting the reduction of macrophage infiltration to colitic tissue (Figure 5B). To evaluate the effect of CEE on pro-inflammatory cytokines, mRNA expression levels of genes encoding IL-6, IL-1β, and TNF-α were determined by qRT-PCR in DSS-treated colonic tissues. Compared with the control group, DSS administration increased the mRNA expression levels of genes encoding IL-6, IL-1β, and TNF-α (Figure 5C). In contrast, CEE treatment (100 or 200 mg/kg) significantly reduced the mRNA expression levels of these cytokines in colonic tissue with those of DSS-treated group. These results demonstrated that CEE treatment reduced the accumulation of neutrophils and macrophages following the inhibition of pro-inflammatory cytokines mRNA expression in DSS-treated mice.

### 3.5. CEE Attenuated the Protein Expression Levels of Cyclooxygenase (COX)-2 and Inducible Nitric Oxide Synthase (iNOS) and the Activation of NF-κB and STAT3 in Colon Tissue from DSS-Treated Mice

**Figure 6:**
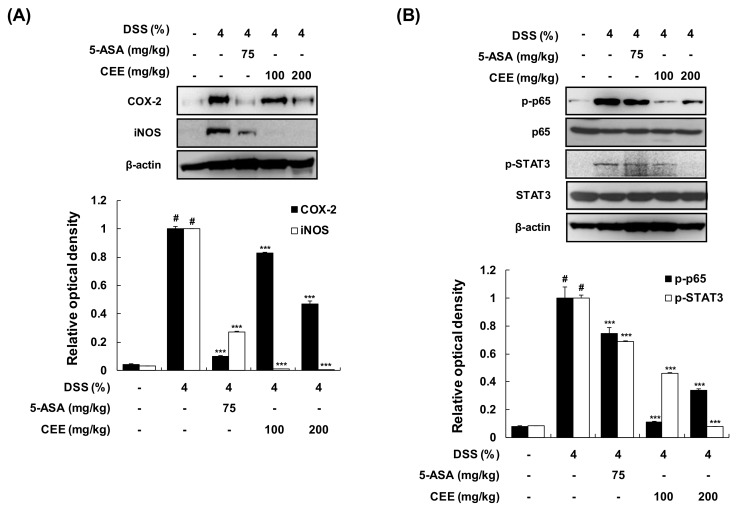
Effects of CEE on inflammation-related proteins and transcription factors in mice with DSS-induced colitis. After administration with or without CEE (100 or 200 mg/kg/day p.o.) for 9 days, whole protein extracts were prepared from DSS-exposed colonic tissues. (**A**, **B**) The protein expression levels of COX-2, iNOS, p-p65, and p-STAT3 were evaluated by western blot analysis. *β*-Actin was used as an internal control. The ratio of relative optical density was calculated by densitometric analysis and normalized to the internal control. Data are presented as the mean ± SD of three independent experiments. *^#^p* < 0.001 vs. the control group; ^***^p < 0.001 vs. the DSS-fed colitis group.

To examine the anti-inflammatory effect of CEE on DSS-treated colonic tissue, the protein expression levels of COX-2 and iNOS and the activation of NF-κB and STAT3 were analyzed using Western blot analysis. Compared with the vehicle-treated control group, the DSS-treated group showed marked upregulation of COX-2 and iNOS protein levels (Figure 6A). However, in the DSS + CEE-treated group (100 or 200 mg/kg), these DSS-induced changes in protein expression were restored to normal levels. Of the multitude of transcription factors involved in intracellular signaling pathways, NF-κB and STAT3 are known to regulate genes involved in the expression of proinflammatory mediators [20,21]. In line with the change of COX-2 and iNOS protein expression, CEE suppressed DSS-induced NF-κB and STAT3 phosphorylation in colonic tissue (Figure 6B). These results indicated that CEE exerted an anti-colitis effect through the suppression of inflammatory protein expression by interfering with the NF-κB and STAT3 activation in colon tissue from DSS-treated mice.

## 4. Discussion

IBD, which includes CD and UC, is a complex and multifactorial disease of the human gastrointestinal tract. Over the past 20 years, several murine models of colitis have been developed to study human IBD mechanistically [10]. In the present study, we demonstrated, for the first time, the anti-inflammatory properties of *C. arietinum* ethanolic extract. This extract was found to inhibit inducible NF-κB and STAT3 activation and the subsequent inductions of pro-inflammatory mediators, and to protect mice from DSS-induced colitis, which is a well-established experimental model for IBD. The most severe model of murine colitis, which most closely resembles human UC [22], results from the administration of 40–50 kDa DSS in drinking water. Animals given DSS exhibited weight loss and signs of loose stool or diarrhea, sometimes with evidence of rectal bleeding [14]. Moreover, DSS-induced intestinal inflammation results in damage to the epithelial monolayer lining in the large intestine, allowing the dissemination of pro-inflammatory intestinal contents (e.g., bacteria and their products) into the abdominal cavity [10]. In our study, we showed that oral administration of CEE significantly lessened the symptoms associated with UC involving weight loss, bloody stool, diarrhea, colon shortening, and histopathological alterations. The gastrointestinal tract is lined with a fragile epithelial cell layer characterized by high turnover. The preservation of intestinal epithelial barrier requires a substantial balance between apoptosis-induced epithelial loss and proliferation-induced new cell generation [10,23]. Our results agree with those of previous studies reporting that this balance shifts towards excessive apoptosis, indicated by elevated cleaved PARP levels during DSS administration, whereas CEE reduced the DSS-induced epithelial cell death. These results suggest that CEE-induced protection of epithelial proliferation may help maintain intestinal barrier function. 

During UC, neutrophils are recruited to the site of inflammation, and they are found within intestinal crypts and at the bases of ulcerations, forming crypt abscesses [23]. Accordingly, neutrophil-associated markers such as MPO are upregulated in active UC and their concentration correlates with the extent of neutrophil infiltration in severely destroyed colonic tissue [24]. In addition, the total number of macrophages increases, including some subpopulations of macrophages that are not normally present in the inflamed intestinal mucosa [12]. Macrophages, together with neutrophils, are capable of inducing intestinal damage by secreting inflammatory mediators including pro-inflammatory cytokines. As expected, our data also revealed that DSS upregulated MPO activity and F4/80 mRNA expression, a macrophage marker, indicating the infiltration of inflammation-regulating cells. Furthermore, mRNA expression of genes encoding cytokines including IL-6, IL-1β, and TNF-α, and protein expression of COX-2 and iNOS were upregulated in the colon tissue of mice with DSS-induced colitis. However, the oral administration of CEE significantly reduced DSS-induced inflammatory infiltrates and upregulated inflammatory mediators, suggesting that CEE might be capable of preventing DSS-induced impairments in the intestinal mucosa. 

NF-κB and STAT3, two major transcription factors, induce the expression of inflammatory mediators including cytokines, COX-2, and iNOS in UC. The activation of these transcription factors is also significantly higher in intestinal epithelial cells from patients with active UC compared with patients with inactive disease or healthy controls, and these levels correlated with colitis severity [1]. The functional interactions between NF-κB and STAT3 enhance inflammation in the intestinal mucosa and have been investigated in other studies [25,26]. In contrast, abrogation of NF-κB and STAT3 activation has been reported to be associated with improvements in intestinal homeostasis in DSS-administrated mice [27]. The present study showed that CEE administration potently attenuated the DSS-mediated increases in the phosphorylation levels of p65 and STAT3 in colonic tissues. Based on the results of this study, it is likely that both NF-κB and STAT3 signaling are involved in the protective effects of CEE on DSS-induced colitis.

The use of medicinal foods or phytochemicals extracted from food and plants has been a major research focus throughout the last decade as potential therapies for IBD, and many research groups have demonstrated remarkable findings [28]. Many natural products, such as plant-derived extracts, antioxidants, and phytochemicals, have reported strong protective effects against IBD due to their ability to regulate inflammation-mediated cytokines and signaling pathways [29,30,31]. In this study, we found that the beneficial effect of CEE on DSS-induced colitis is related to inhibition of inflammatory mediators by inactivating NF-κB and STAT3 signaling, and our results suggest a new method for treating or preventing UC.

## Figures and Tables

**Table 1:** Evaluation of DAI.

DAI Score	Weight Loss (%)	Stool Consistency	Hematochezia
0	None	Normal	No bleeding
1	1–5		
2	5–10	Loose stools	Slight bleeding
3	10–20		
4	>20	Diarrhea	Gross bleeding

**Table 2:** List of primer sequences for qRT-PCR.

Gene		Sequence
*F*4/80	Forward	AGGACTGGAAGCCCATAGCCAA
	Reverse	GCATCTAGCAATGGACAGCTG
*IL-*6	Forward	GAGGATACCACTCCCAACAGACC
	Reverse	AAGTGCATCATCGTTGTTCATACA
*IL*-1*β*	Forward	ACCTGCTGGTGTGTGACGTT
	Reverse	TCGTTGCTTGGTTCTCCTTG
*TNF-* *α*	Forward	AGCACAGAAAGCATGATCCG
	Reverse	CTGATGAGAGGGAGGCCATT
*β-actin*	Forward	ATCACTATTGGCAACGAGCG
	Reverse	ATCACTATTGGCAACGAGCG

**Table 3:** Retention time (Rt), precursor ion, molecule formula, and ultraviolet Maxima (λ max) of identified peaks of *C. arietinum*.

Compound	Rt (min)	Precursor Ion *(m/z)*	Molecular Formula	λ Max (nm)
1. **adenosine**	22.93	268.10628 [M + H]^+^	C_10_H_14_N_5_O_4_	258
2. **guanine**	24.03	152.04912 [M + H]^+^	C_5_H_6_N_5_O	254
3. **guanosine**	25.05	284.09571 [M + H]^+^	C_10_H_14_N_5_O_5_	252
4. **tryptophan**	42.62	205.10663 [M + H]^+^	C_11_H_13_N_2_O_2_	219,249
